# Safety of transarterial chemoembolization on renal function in combined hepatocellular carcinoma and chronic kidney disease patients

**DOI:** 10.1002/kjm2.12925

**Published:** 2024-12-30

**Authors:** Zu‐Yau Lin, Ming‐Lun Yeh, Po‐Cheng Liang, Chung‐Feng Huang, Jee‐Fu Huang, Chia‐Yen Dai, Ming‐Lung Yu, Wan‐Long Chuang

**Affiliations:** ^1^ Division of Hepatobiliary Medicine, Department of Internal Medicine Kaohsiung Medical University Hospital Kaohsiung Taiwan; ^2^ Department of Internal Medicine, Faculty of Medicine, College of Medicine Kaohsiung Medical University Kaohsiung Taiwan; ^3^ Department of Internal Medicine, Faculty of Post‐Baccalaureate Medicine, College of Medicine Kaohsiung Medical University Kaohsiung Taiwan; ^4^ Center for Cancer Research Kaohsiung Medical University Kaohsiung Taiwan; ^5^ Center for Liquid Biopsy and Cohort Research Kaohsiung Medical University Kaohsiung Taiwan

**Keywords:** acetylcysteine, chronic renal disease, Cockcroft‐Gault glomerular filtration rate, contrast‐induced nephropathy, liver cirrhosis

## Abstract

This study was to investigate the safety of transarterial chemoembolization (TACE) which required injection of contrast medium on renal function in combined hepatocellular carcinoma and chronic kidney disease (CKD) patients. A total of 265 patients admitted for the first session of TACE were included for analysis. CKD was defined as Cockcroft‐Gault glomerular filtration rate (CG‐GFR) < 60 mL/min/1.73 m^2^. The odds ratio (OR) and 95% confident interval (CI) were calculated to show the influence of factors on renal function. Overall, 24.07% patients with CKD and 31.21% patients without CKD showed exacerbated renal function at discharge. However, 73.15% patients with CKD and 63.69% patients without CKD showed significantly improved renal function (all *p* = 0.00001). No significant difference in influence of TACE on renal function between patients with and without CKD (*p* = 0.20509). Factors to exacerbate the serum creatinine level at the third day after TACE included proteinuria ≥1+ (OR 2.2469, 95% CI = 1.1559–4.3675) and glycated hemoglobin ≥7% (OR 2.0796, 95% CI = 1.0497–4.1200). These factors could be obliterated by admission for more than 3 days after TACE. Serum albumin level <3 g/dL at admission was the only factor to exacerbate renal function at discharge (OR 4.4179, 95% CI = 1.3964–13.9776). In conclusion, TACE exerted same influence on renal function between patients with and without CKD. Most patients showed improved renal function at discharge. Low serum albumin level, proteinuria and poor diabetes mellitus control were factors to exacerbate renal function after TACE.

## 1 INTRODUCTION

Transarterial chemoembolization (TACE) is a well‐established standard treatment for intermediate‐stage hepatocellular carcinoma (HCC).^1^ This treatment requires injection of contrast medium for angiographic location of target for embolization. Contrast‐induced nephropathy (CIN) is an iatrogenic acute kidney injury characterized by progressive decline in kidney function within a few days of contrast medium administration.^2^, ^3^, ^4^, ^5^, ^6^ Low glomerular filtration rates, diabetes mellitus (DM), congestive heart failure, intraarterial intervention, higher volume of contrast, volume depletion, old age, multiple myeloma, hypertension, and hyperuricemia had been described to increase the prevalence of CIN.^2^, ^5^, ^6^ Chronic hepatitis B and C virus infection, alcohol and metabolic associated fatty liver disease (MAFLD)^7^, ^8^ are important risk factors for HCC.^9^ Hepatitis B and C related glomerulonephritis as well as IgA nephropathy are well established extra‐hepatic manifestations in patients with chronic liver disease.^10^ The diagnostic criteria for MAFLD are based on evidence of hepatic steatosis, in addition to one of the following three criteria, namely overweight/obesity, presence of type 2 DM, or evidence of metabolic dysregulation.^8^ This indicates that MAFLD has the potential to involve renal damage. The renal function in patients with HCC treated by TACE are thus in risk to induce CIN. Patients with chronic kidney disease (CKD) may hesitate to receive TACE for fear of further exacerbation of renal function even progression to end stage renal disease due to CIN. However, the real‐word safety of TACE on renal function in combined hepatocellular carcinoma and chronic kidney disease patients has not been well clarified. Understanding the impact of TACE on renal function and the influence factors in patients with HCC can provide critical information for the treatment of these patients. The purposes of this study were to elucidate these issues.

## 2 PATIENTS AND METHODS

From January 2017 to January 2023, a total of 265 consecutive patients admitted to our institution to receive the first session of TACE for the treatment of HCC were enrolled for this retrospective study. Among these patients, 242 patients were newly diagnosed HCC and the remaining 23 patients were previously treated by other methods for HCC. Patients presenting life‐threating acute illness such as cerebral or cardiovascular infarction, infection, gastrointestinal hemorrhage or rupture of HCC and patients under hemodialysis or peritoneal dialysis were excluded. The personal past medical history, admission records and the laboratory data from day of admission till the first time visit in outpatient department (OPD) after discharge were collected for analysis. Since all patients needed fasting for at least 8 h before TACE and usually suffered from abdominal discomfort after TACE leading to decreased appetite, routine intravenous hydration before and after TACE was applied to all patients. No particularly fixed hydration protocol was applied to these patients. The basic rule to determine the amount of intravenous hydration was based on the individual condition of the patient. All patients received complete angiographic examination from celiac trunk, superior mesenteric artery, right hepatic artery and left hepatic artery to locate the target for embolization. Nonionic, water‐soluble Ultravist®‐370 (Iopromide with iodine 370 mg/mL, Bayer Taiwan Company Ltd. 53 & 54F, No. 7, Sec. 5 Xinyi Road Taipei 101, Taiwan, ROC) was applied as contrast medium for angiography. The total amount of injected contrast medium in each session of TACE ranged from 50 to 70 mL. Embolization of HCC was accomplished by either conventional method (cTACE) or using drug‐eluting microspheres (DEMs). The applied DEMs included DC Bead™ (Boston Scientific Corporation, 300 Boston Scientific Way, Marlborough, MA 01752‐1234, USA), HepaSphere™ (Merit Medica, 1600 West Merit Parkway, South Jordan, UT 84095, USA) or TANDEM™ (CeloNova BioSciences, Inc. San Antonio, CE 0086, USA). The embolized chemotherapeutic agent was epirubicin. Part of patients in cTACE group also received mitomycin C. The adverse events of TACE were recorded based on the Common Terminology Criteria for Adverse Events (CTCAE) Version 5.0 published at November 27, 2017. The American Joint Committee on Cancer (AJCC) TNM staging system 8th edition was applied for tumor staging. Evaluation of renal function was based on serum creatinine level to calculate the Cockcroft‐Gault glomerular filtration rate (CG‐GFR). CKD was defined as CG‐GFR < 60 mL/min/1.73 m^2^. This study was approved by the Institutional Review Board.

### 2.1 Statistical analysis

The Mann–Whitney *U* test, *t*‐test, Fisher exact test and chi‐square test were applied for the calculation the significant difference between two groups. The non‐parametric sign test was used to evaluate the results of a repeated‐measures study. The odds ratio (OR) and 95% confident interval (CI) were calculated to show the influence of factors on renal function. The statistical significance was defined as *p* < 0.05.

## 3 RESULTS

### 3.1 Characteristics of patients and adverse events of TACE

Table 1 shows the characteristics of patients and adverse events of TACE. The lowest CG‐GFR in this study was 9.96 mL/min/1.73 m^2^. Total intravenous fluid injection at day of TACE ranged from 500 to 3350 mL with the median of 1150 mL. Among patients using DEMs, DC Bead™ was applied in 23 patients, HepaSphere™ in 20 patients and TANDEM™ in the remaining 22 patients. Patient received DEMs showed significantly larger embolized tumor size (*p* = 0.002717) and higher dose of embolized epirubicin (*p* < 0.00001) than cTACE group. However, there was no significant difference in duration of admission and adverse events between cTACE and DEMs groups. Overall, two patients (0.75%) expired during admission. One patient (T3N0M0, stage IIIA, embolized tumor size 12.8 cm) combined with congestive heart failure expired 4 days after TACE due to acute myocardial infarction complicated with cardiogenic shock and lactic acidosis. Another one patient (T4N1M0, stage IVA, embolized tumor size 13.5 cm) expired 23 days after TACE due to rupture of esophageal varices. Although both patients belonged to DEMs group, there was no significant difference in mortality during admission between cTACE group and DEMs group (*p* = 0.0595). Patients with embolized tumor size ≥5 cm showed significantly longer admission days (7.8 ± 4.5 days vs. 5.1 ± 2.39 days, *p* < 0.00001) and higher incidence of ≥grade 3 adverse events (*p* = 0.002498) than patients with embolized tumor size <5 cm. None of the patients suffered from ≥grade 3 adverse event of increase in serum creatinine level during admission.

**TABLE 1:** Characteristics of 265 patients with hepatocellular carcinoma and adverse events of TACE.

	Total	cTACE	TACE using DEMs	*p* Value
(A) Characteristics of patients	265	200	65	
Age (year)	40–99, 68	40–99, 67.5	41–92, 68	0.238
Sex (male/female)	173/92	133/67	40/25	0.46542
Hypertension (−/+)	111/154	83/117	28/37	0.0501
Diabetes mellitus (−/+)	157/108	117/83	40/25	0.1876
HbA1c (%)				
<7	224	168	56	0.120947
7–8	24	16	8	
>8	17	16	1	
LC (−/+)	106/159	76/124	30/35	0.243715
(a) No LC or LC Child‐Pugh class A	241	181	60	0.8059
(b) LC Child‐Pugh class B	24	19	5	
CG‐GFR (mL/min/1.73 m^2^)				
≥60	157	120	37	0.428327
45–59	53	42	11	
<45	55	38	17	
Proteinuria (negative/≥1+)	221/44	165/35	56/9	0.491618
Etiologies of hepatocellular carcinoma				NA
CHB/CHC/alcohol/CHB + CHC/CHB + CHD/CHB + alcohol/CHC + alcohol/MAFLD/unknown	90/75/16/4/1/19/12/7/41	67/59/15/1/0/16/11/4/27	23/16/1/3/1/3/1/3/14	
AJCC tumor staging^a^				
I	67	48	19	0.103778
II	81	68	13	
≥III	117	84	33	
Embolized tumor size				
<5 cm	122	100	22	0.002717
5–10 cm	88	68	20	
>10 cm	55	32	23	
Embolized epirubicin (mg)	1.7–180, 20	1.7–150, 18.45	2.5–180, 88	<0.00001
Duration of admission (day)	3–29, 6	3–22, 6	3–29, 6	0.09492
Total IV fluid at day of TACE (mL)	500–3350, 1150	500–3000, 1075	500–3350, 1400	0.1443
(B) Adverse events of TACE^b^				
Blood bilirubin increased				
None	79	63	16	0.653567
Grade 1	116	87	29	
Grade 2	65	46	19	
Grade 3	5	4	1	
Alanine aminotransferase increased				
None	31	25	6	0.792236
Grade 1	121	90	31	
Grade 2	55	43	12	
Grade 3 and 4^c^	58	42	16	
Blood creatinine increased				
None	223	164	59	0.092604
Grade 1 and 2^d^	42	36	6	
Anemia				
None	59	47	12	0.106364
Grade 1	135	101	34	
Grade 2	67	51	16	
Grade 3	4	1	3	
Upper gastrointestinal Hemorrhage				
None	261	198	63	0.2529
Grade 3	4	2	2	
Esophageal hemorrhage				
None	264	200	64	0.2453
Grade 5	1	0	1	
Bacteremia				
None	264	199	65	1
Grade 2	1	1	0	
Sepsis				
None	264	199	65	1
Grade 3	1	1	0	
Lung infection				
None	264	199	65	1
Grade 3	1	1	0	
Pleural effusion				
None	264	199	65	1
Grade 2	1	1	0	
Ascites				
None	263	199	64	0.4311
Grade 2	2	1	1	
Heart failure				
None	264	200	64	0.2453
Grade 5	1	0	1	
Hypokalemia				
None	263	198	65	1
Grade 3	2	2	0	

*Note*: Seventy‐two patients in cTACE group also combined with mitomycin C ranging from 0.4–6 mg with median dose of 4 mg. Proteinuria was determined by the urine protein dipstick test. All patients suffered from only grade 1 fever. Data for continuous variables were expressed as range and median. The Mann–Whitney *U* test, Fisher exact test and chi‐square test were applied for the calculation the significant difference between two groups. *p* Value was calculated to show the statistical difference between cTACE group and TACE using DEMs group.

Abbreviations: CHB, chronic hepatitis B; CHC, chronic hepatitis C; CHD, chronic hepatitis D; cTACE, conventional TACE; DEMs, drug‐eluting microspheres; G‐GFR, Cockcroft‐Gault glomerular filtration rate; HbA1c, glycated hemoglobin; IV, intravenous; LC, liver cirrhosis; MAFLD, metabolic associated fatty liver disease; NA, not applicable; TACE, transarterial chemoembolization.

^a^ The American Joint Committee on Cancer (AJCC) TNM staging system 8th edition was applied for tumor staging.

^b^ The adverse events were based on the Common Terminology Criteria for Adverse Events (CTCAE) Version 5.0 published at November 27, 2017.

^c^ Two patients in cTACE group suffered from grade 4 adverse events.

^d^ Two patients in DEMs group suffered from grade 2 adverse events.

### 3.2 Serial changes in renal function

**FIGURE 1:**
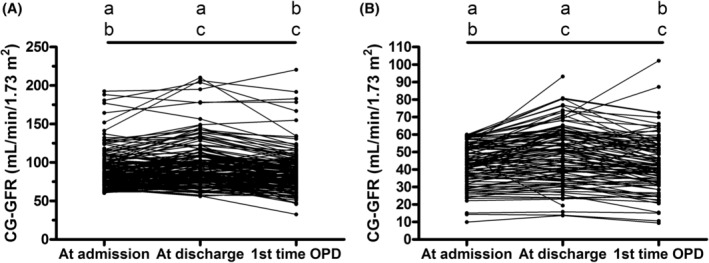
Changes of CG‐GFR from admission to the first time OPD. (A) patients with CG‐GFR ≥ 60 mL/min/1.73 m^2^, a: *p* = 0.00001, b: *p* = 0.1146, c: *p* < 0.0001. (B) patients with CG‐GFR < 60 mL/min/1.73 m^2^, a: *p* = 0.00001, b: *p* = 0.76067, c: *p* < 0.0001. Only 251 patients had the first time OPD data for comparison. Sign test was applied for the statistical analysis. CG‐GFR, Cockcroft‐Gault glomerular filtration rate; OPD, Outpatient department.

Table 2 and Figure 1 show serial changes in renal function from admission to the first time OPD. Overall, 73.15% patients with CKD (*p* = 0.00001) and 63.69% patients without CKD (*p* = 0.00001) showed significantly improved renal function at discharge compared to the data collected at admission. However, the data collected at the first time OPD in patients either with or without CKD showed significantly exacerbated renal function compared to discharge (75.76%, *p* < 0.00001; 69.08%, *p* < 0.00001, respectively) but no significant difference with data at admission. The serum creatinine levels detected at the third day after TACE in patients either with or without CKD showed significant improvement compared to the data collected at admission (70.75%, *p* = 0.00002; 58.71%, *p* = 0.00414, respectively). Patients either with or without CKD also showed further significant improvement in serum creatinine levels at discharge compared to the data collected at the third day after TACE (64.29%, *p* = 0.04233; 62.96%, *p* = 0.0265, respectively). Overall, 27.36% patients with CKD and 36.77% patients without CKD showed increased serum creatinine levels at the third day after TACE. Admission for more than 3 days after TACE decreased the serum creatinine levels in part of the patients and finally 24.07% patients with CKD and 31.21% patients without CKD showed exacerbated renal function at discharge.

**TABLE 2:** Serial changes in renal function from admission to the first time outpatient department.

	Improvement	Exacerbation	No change	Total	*p* Value
(A) CG‐GFR (mL/min/1.73 m^2^)					
(1) Discharge vs. admission	179	75	11	265	<0.00001
(a) CG‐GFR ≥ 60	100	49	8	157	0.00001
(b) CG‐GFR < 60	79	26	3	108	0.00001
(2) First time OPD vs. admission^a^	113	129	9	251	0.30371
(a) CG‐GFR ≥ 60	63	82	7	152	0.1146
(b) CG‐GFR < 60	50	47	2	99	0.76067
(3) First time OPD vs. discharge	65	180	6	251	<0.00001
(a) CG‐GFR ≥ 60	42	105	5	152	<0.00001
(b) CG‐GFR < 60	23	75	1	99	<0.00001
(B) Creatinine level (mg/dL)					
(1) Third day after TACE vs. admission^b^	166	86	9	261	<0.00001
(a) CG‐GFR ≥ 60	91	57	7	155	0.00414
(b) CG‐GFR < 60	75	29	2	106	0.00002
(2) Third day after TACE vs. discharge^c^	61	32	3	96	0.00264
(a) CG‐GFR ≥ 60	34	18	2	54	0.0265
(b) CG‐GFR < 60	27	14	1	42	0.04233

*Note*: Sign test was applied for the statistical analysis.

Abbreviations: CG‐GFR, Cockcroft‐Gault glomerular filtration rate; OPD, outpatient department; TACE, transarterial chemoembolization.

^a^ Fourteen patients did not perform renal function examination at the first time OPD after discharge.

^b^ Four patients did not perform creatinine determination at the third day after TACE.

^c^ Except for the third day creatinine examination, 96 patients admitted for more than 3 days had another creatinine data at time of discharge.

### 3.3 Factors to influence the renal function

Table 3 shows the odds ratio of factors to increase the serum creatinine level at the third day after TACE. Patients with proteinuria ≥1+ showed the OR of 2.2469 (95% CI = 1.1559 to 4.3675, *p* = 0.0170) and DM patients with glycated hemoglobin (HbA1c) ≥ 7% showed the OR of 2.0796 (95% CI = 1.0497 to 4.1200, *p* = 0.0358) to increase the creatinine levels at the third day after TACE. On the contrary, the OR of 0.4502 (95% CI = 0.2191 to 0.9253, *p* = 0.0299) to increase serum creatinine level at the third day after TACE were detected in patients with CG‐GFR < 45 mL/min/1.73 m^2^. Exclusion the patients with proteinuria ≥1+, DM patients with HbA1c ≥ 7% showed the OR of 2.9375 (95% CI = 1.2598 to 6.8495, *p* = 0.0126) and DM patients with HbA1c ≥ 7.5% showed the OR of 2.6296 (95% CI = 1.0771 to 6.4199, *p* = 0.0337) to increase the serum creatinine levels at the third day after TACE. On the contrary, patients with CG‐GFR < 60 mL/min/1.73 m^2^ showed the OR of 0.3875 (95% CI = 0.1946 to 0.7718, *p* = 0.0070) and patients with CG‐GFR < 45 mL/min/1.73 m^2^ showed the OR of 0.0668 (95% CI = 0.0089 to 0.5017, *p* = 0.0085) to increase serum creatinine level at the third day after TACE (Table S1). Table 4 shows the OR of factors to exacerbate renal function at discharge compared to the data at admission. Only serum albumin level <3 g/dL at admission was the factor to exacerbate renal function at discharge (OR 4.4179, 95% CI = 1.3964 to 13.9776, *p* = 0.0115). Table 5 shows the OR of factors to exacerbate renal function at the 1st time OPD after discharge compared to the data at discharge. AJCC tumor staging ≥III (OR 0.3345, CI = 0.1891 to 0.5916, *p* = 0.0002), embolized tumor size ≥5 cm (OR 0.3929, CI = 0.2183 to 0.7071, *p* = 0.0018), adverse events of TACE ≥Grade 3 (OR 0.4530, CI = 0.2511 to 0.8170, *p* = 0.0085) and admission days ≥7 days (OR 0.5306, CI = 0.3031 to 0.9290, *p* = 0.0266) showed as benefit factors to continue improvement in renal function detected at the first time OPD compared to discharge.

**TABLE 3:** Odds ratio of factors to exacerbate the serum creatinine level at the third day after TACE.

	Serum creatinine (mg/dL)^a^		Odds ratio	95% CI	*p* Value
	Increase (*n* = 86)	Decrease or no change (*n* = 175)			
(A) Factors at admission					
Hypertension (+/−)	57/29	95/80	1.6552	0.9675–2.8317	0.0659
Diabetes mellitus (+/−)	39/47	67/108	1.3376	0.7931–2.2558	0.2754
HbA1c (%) ≥ 7 (+/−)	19/67	21/154	2.0796	1.0497–4.1200	0.0358
HbA1c (%) ≥ 7.5 (+/−)	14/72	17/158	1.8072	0.8449–3.8654	0.1271
HbA1c (%) ≥ 8 (+/−)	7/79	13/162	1.1042	0.4239–2.8764	0.8392
Liver cirrhosis (+/−)	53/33	103/72	1.1227	0.6617–1.9049	0.6679
Liver cirrhosis Child‐Pugh class B (+/−)	10/76	14/161	1.5132	0.6428–3.5620	0.3430
Serum albumin <3 g/dL (+/−)	7/79	6/169	2.4958	0.8121–7.6698	0.1103
Hemoglobin <10 g/dL (+/−)	14/72	29/146	0.9789	0.4873–1.9664	0.9523
CG‐GFR (mL/min/1.73 m^2^) < 60 (+/−)	29/57	77/98	0.6475	0.3783–1.1084	0.1131
CG‐GFR (mL/min/1.73 m^2^) < 45 (+/−)	11/75	43/132	0.4502	0.2191–0.9253	0.0299
Proteinuria ≥1+ (+/−)	21/65	22/153	2.2469	1.1559–4.3675	0.0170
AJCC tumor staging ≥ III (+/−)	45/41	71/104	1.6077	0.9561–2.7034	0.0734
(B) Factors within 3 days after TACE					
Embolized tumor size ≥5 cm (+/−)	52/34	89/86	1.4779	0.8750–2.4961	0.1441
TACE using DEMs (+/−)	22/64	41/134	1.1235	0.6181–2.0420	0.7025
Total IV fluid at day of TACE <1000 mL (+/−)	6/80	8/167	1.5656	0.5256–4.6638	0.4209
Total IV fluid within 3 days after TACE <1000 mL (+/−)	3/83	7/168	0.8675	0.2187–3.4407	0.8397
CG‐GFR (mL/min/1.73 m^2^) < 60 patients treated with NAC^b^ (*n* = 106) (+/−)	10/21	30/45	0.7143	0.2952–1.7281	0.4554

Abbreviations: AJCC, The American Joint Committee on Cancer; CG‐GFR, Cockcroft‐Gault glomerular filtration rate; CI, confidence interval; cTACE, conventional TACE; DEMs, drug‐eluting microspheres; HbA1c, glycated hemoglobin; IV, intravenous; NAC, N‐acetylcysteine; TACE, transarterial chemoembolization.

^a^ Four patients did not have serum creatinine data at the third day after TACE.

^b^ The patients took effervescent NAC 600 mg twice per day from day of admission till discharge.

**TABLE 4:** Odds ratio of factors to exacerbate renal function at discharge compared to the data at admission.

	CG‐GFR (mL/min/1.73 m^2^)		Odds ratio	95% CI	*p* Value
	Decrease (*n* = 75)	Increase or no change (*n* = 190)			
(A) Factors at admission					
Hypertension (+/−)	44/31	110/80	1.0323	0.6001–1.7756	0.9087
Diabetes mellitus (+/−)	36/39	72/118	1.5128	0.8820–2.5949	0.1326
HbA1c (%) ≥ 7 (+/−)	16/59	25/165	1.7898	0.8938–3.5841	0.1004
HbA1c (%) ≥ 7.5 (+/−)	13/62	18/172	2.0036	0.9275–4.3282	0.0770
HbA1c (%) ≥ 8 (+/−)	7/68	13/177	1.4016	0.5364–3.6622	0.4909
Liver cirrhosis (+/−)	47/28	112/78	1.1690	0.6745–2.0262	0.5779
Liver cirrhosis Child‐Pugh class B (+/−)	10/65	14/176	1.9341	0.8185–4.5702	0.1327
Serum albumin <3 g/dL (+/−)	8/67	5/185	4.4179	1.3964–13.9776	0.0115
Hemoglobin <10 g/dL (+/−)	15/60	28/162	1.4464	0.7229–2.8941	0.2969
CG‐GFR (mL/min/1.73 m^2^) < 60 (+/−)	26/49	82/108	0.6989	0.4010–1.2180	0.2061
CG‐GFR (mL/min/1.73 m^2^) < 45 (+/−)	11/64	44/146	0.5703	0.2767–1.1753	0.1280
Proteinuria ≥1+ (+/−)	17/58	27/163	1.7695	0.8993–3.4816	0.0984
AJCC tumor staging ≥ III (+/−)	38/37	79/111	1.4430	0.8436–2.4684	0.1805
(B) Factors during admission					
Embolized tumor size ≥5 cm (+/−)	42/33	101/89	1.1215	0.6551–1.9200	0.6759
TACE using DEMs (+/−)	16/59	49/141	0.7804	0.4111–1.4814	0.4482
Grade 3 to Grade 5 adverse events of TACE (+/−)	20/55	49/141	1.0464	0.5707–1.9186	0.8835
Admission days ≥7 days (+/−)	31/44	66/124	1.3237	0.7652–2.2899	0.3160

Abbreviations: AJCC, The American Joint Committee on Cancer; CG‐GFR, Cockcroft‐Gault glomerular filtration rate; CI, confidence interval; cTACE, conventional TACE; DEMs, drug‐eluting microspheres; GPT, alanine aminotransferase; HbA1c, glycated hemoglobin; TACE, transarterial chemoembolization.

**TABLE 5:** Odds ratio of factors to exacerbate renal function at the first time OPD after discharge compared to the data at discharge.

	CG‐GFR (mL/min/1.73 m^2^)^a^		Odds ratio	95% CI	*p* Value
	Decrease (*n* = 180)	Increase or no change (*n* = 71)			
(A) Factors at admission					
Hypertension (+/−)	110/70	36/35	1.5278	0.8784–2.6571	0.1334
Diabetes mellitus (+/−)	70/110	34/37	0.6925	0.3981–1.2048	0.1934
HbA1c (%) ≥ 7 (+/−)	25/155	15/56	0.6022	0.2962–1.2240	0.1610
HbA1c (%) ≥ 7.5 (+/−)	19/161	12/59	0.5802	0.2655–1.2681	0.1724
HbA1c (%) ≥ 8 (+/−)	13/167	7/64	0.7117	0.2717–1.8643	0.4888
Liver cirrhosis (+/−)	110/70	41/30	1.1498	0.6580–2.0093	0.6240
Liver cirrhosis Child‐Pugh class B (+/−)	18/162	5/66	1.4667	0.5229–4.1137	0.4667
CG‐GFR (mL/min/1.73 m^2^) < 60 (+/−)	75/105	24/47	1.3988	0.7878–2.4837	0.2519
CG‐GFR (mL/min/1.73 m^2^) < 45 (+/−)	38/142	14/57	1.0895	0.5489–2.1625	0.8063
Proteinuria ≥1+ (+/−)	28/152	13/58	0.8219	0.3984–1.6953	0.5954
AJCC tumor staging ≥ III (+/−)	66/114	45/26	0.3345	0.1891–0.5916	0.0002
(B) Factors during admission					
Embolized tumor size ≥5 cm (+/−)	87/93	50/21	0.3929	0.2183–0.7071	0.0018
TACE using DEMs (+/−)	49/131	12/59	1.8391	0.9113–3.7113	0.0890
Adverse events of TACE ≥ Grade 3 (+/−)	41/139	28/43	0.4530	0.2511–0.8170	0.0085
Admission days ≥7 days (+/−)	59/121	34/37	0.5306	0.3031–0.9290	0.0266
(C) Factors at the first time OPD^b^					
Hemoglobin <10 g/dL (+/−)	23/139	10/55	0.9101	0.4067–2.0364	0.8186
Serum albumin <3 g/dL (+/−)	8/125	1/54	3.4560	0.4218–28.3142	0.2478
Total bilirubin >2 mg/dL (+/−)	16/151	2/68	3.6026	0.8058–16.1074	0.0935

Abbreviations: AJCC, The American Joint Committee on Cancer; CG‐GFR, Cockcroft‐Gault glomerular filtration rate; CI, confidence interval; DEMs, drug‐eluting microspheres; HbA1c, glycated hemoglobin; OPD, outpatient department; TACE, transarterial chemoembolization.

^a^ Fourteen patients did not perform renal function examination at the first time OPD after discharge.

^b^ Only 227 patients had available hemoglobin levels, 188 patients had available serum albumin levels, and 237 patients had available serum bilirubin levels at the first time OPD after discharge.

## 4 DISCUSSION

TACE can be carried out by either cTACE or using DEMs. Although the embolized tumor size and the dose of embolized epirubicin in DEMs group were significantly larger than cTACE group, there was no significant difference in the occurrence of adverse events between cTACE and DEMs groups. This suggests that there was no significant difference in the effect of kidney function between the two groups. Therefore, the data from both groups could be combined for further analysis.

Acute renal damage in HCC patients receiving TACE can be caused by tumor lysis syndrome, nephrotoxic effects of chemotherapeutic agent or CIN. Tumor lysis syndrome results from massive tumor cell lysis with sudden and rapid release of nuclear and cytoplasmic degradation products of malignant cells leading to severe alterations in the metabolic profile.^11^, ^12^, ^13^, ^14^, ^15^ Acute kidney injury due to tumor lysis is potentiated by the precipitation of uric acid and calcium phosphate as well as renal vasoconstriction.^12^ Hepatocellular carcinoma is the most frequently reported solid tumor complicated this syndrome after treatment^11^, ^13^, ^15^ especially after TACE.^13^ Only two patients in DEMs group suffered from grade 2 adverse event of increase in serum creatinine level. Therefore, tumor lysis syndrome could not be the explanation for the exacerbation of renal function caused by TACE in the present study. Mitomycin C is a well‐known dose‐dependent nephrotoxic drug occurring at cumulative dose levels of 30 mg/m^2^ or more.^16^ The applied mitomycin C doses in 72 cTACE group patients were far low away from this level and none of patients in cTACE group showed larger than grade 1 adverse event of increase in serum creatinine level during admission. Therefore, CIN was the most possible explanation for the exacerbation of renal function caused by TACE in the study. The possible mechanisms of CIN include direct nephrotoxic effect of contrast media on tubular epithelial cells, release of vasoactive molecules, intrarenal vasoconstriction, excessive production of reactive oxygen species and epigenetic regulation in contrast‐induced nephropathy.^3^, ^4^, ^5^, ^6^ Adequate personal hydration can reduce the occurrence of CIN.^3^, ^4^ The incidence of exacerbation of renal function caused by TACE detected at discharge in the present study was around 24%–32%. On the contrary, patients either with or without CKD showed significantly improved renal function at discharge. Moreover, prolonged admission for more than 3 days after TACE could further significantly improve the renal function in part of the patients either with or without CKD. These results indicate that adequate intravenous hydration from day of TACE combined with proper individual medical care during admission was an efficient way to prevent exacerbation or to improve the renal function in patients receiving TACE including those with CKD. Improvement of renal function at the third days after TACE simply by intravenous hydration especially in patients with low CG‐GFR suggests that the impaired renal function at admission in these patients could be partially explained by insufficient effective blood volume. However, patients either with or without CKD showed significantly exacerbated renal function at the first time OPD. On the other hand, AJCC tumor staging ≥III, embolized tumor size ≥5 cm, adverse events of TACE ≥grade 3 and admission days ≥7 days became benefit factors to continue improvement in renal function after discharge. These factors were closely related. In comparison with patients with AJCC tumor staging <III or tumor size <5 cm, patients with AJCC tumor staging ≥III or tumor size ≥5 cm usually showed more severe adverse events of TACE and admitted for longer duration to receive intensive care. These patients were allowed to discharge only at their very stable condition. This may explain why the renal function in these patients could continue to improve after discharge. These findings pointed out the important issues including timing for discharge and education for adequate daily hydration and self‐care to prevent re‐exacerbation of renal function after discharge.

Serum albumin is the main plasma protein to maintain the intravascular oncotic pressure to oppose both the intravascular hydrostatic blood pressure and the interstitial colloidal osmotic pressure.^17^, ^18^, ^19^ Low serum albumin level not only can precipitate accumulation of excess extravascular fluid but also can resulting insufficient effective blood volume which can further exacerbate renal function. TACE can cause various degrees of adverse effects such as decrease in appetite, abdominal discomfort, hepatic damage and fever which can further decrease serum albumin level due to decrease in oral intake and/or hepatic synthesis. The effective blood volume may become insufficient and thus to exacerbate the renal function. Intravenous hydration without keeping adequate serum albumin level is unable to correct insufficient effective blood volume and can induce accumulation of fluid in extravascular space. Therefore, low serum albumin level at admission became the unfavored factor to exacerbate renal function at discharge. Low serum albumin level at admission was not an exacerbated factor for renal function at the third day after TACE might be due to short comparative interval and other factors including proteinuria and poor DM control to mask its influence. Proteinuria indicates the presence of renal damage and the possibility of hypoalbuminemia. Poor DM control also indicates combined chronic kidney injury.^20^, ^21^ Injection of contrast medium in these patients can further induce renal damage.^2^, ^5^, ^6^ This can explain by why proteinuria and poor DM control increased serum creatinine level at the third day after TACE. However, these unfavored factors could be obliterated during further admission for more than 3 days. This again emphasize the importance of proper medical care during admission.

N‐acetylcysteine is an antioxidant which has been described for the potential of protection against CIN. Although the patient number was not large, the present study did not show significant benefit of N‐acetylcysteine to prevent exacerbation of renal function after TACE in patients with low CG‐GFR. This result was in accordance with the previous reports.^22^

The weakness of the present study was data originated from only one medical center. Moreover, most HCC were caused by chronic viral hepatitis rather than MAFLD or alcohol. Further studies using predominant MAFLD or alcohol induced HCC are required to complement our results. On the other hand, the non‐parametric sign test rather than paired t test was applied for statistical calculation because several data were markedly skewed. The reason to collect data form patients who underwent TACE for the first time rather than multiple TACE from the same patients was to minimize statistical bias due to individual effects.

In conclusion, TACE exerted same influence on renal function between patients with and without CKD. Low serum albumin level, proteinuria and poor diabetes mellitus control were factors to exacerbate renal function after TACE. On the contrary, most patients either with or without CKD showed improved renal function at discharge which pointed out the importance of medical care and timing for discharge. For the prevention of re‐exacerbation of renal function after discharge, personal education for adequate daily hydration and self‐care were mandatory.

## CONFLICT OF INTEREST STATEMENT

The authors declare no conflict of interest.

## ETHICS STATEMENT

This study was approved by the Institutional Review Board (Kaohsiung Medical University Chung‐Ho Memorial Hospital, KMUHIRB‐E(I)‐20240274). Waiver of informed consent for this study was approved by the Institutional Review Board of the hospital.

## Supporting information


Table S1

Appendix S1

## Data Availability

Data sharing is not applicable to this article as no new data were created or analyzed in this study.
