# Supplementary material for: Safety of transarterial chemoembolization on renal function in combined hepatocellular carcinoma and chronic kidney disease patients
Source: Kaohsiung J Med Sci. 2024 Dec 30;41(2):e12925. doi: 10.1002/kjm2.12925 (PMC11827540; doi:10.1002/kjm2.12925)
Supplement: Supplementary file 1 — Table S1 [file KJM2-41-e12925-s002.docx]

**Table 1 Odds ratio of factors to exacerbate the serum creatinine level at the third day after TACE after exclusion patients with proteinuria > 1+**

|  | Serum creatinine (mg/dL)† | | Odds ratio | 95 % CI | *P* value |
| --- | --- | --- | --- | --- | --- |
|  | Increase (n = 65) | Decrease or no change (n = 153) |  |  |  |
| (A) Factors at admission |  |  |  |  |  |
| Hypertension | 40/25 | 76/77 | 1.6211 | 0.8970 to 2.9294 | 0.1096 |
| Diabetes mellitus (+/-) | 24/41 | 51/102 | 1.1707 | 0.6389 to 2.1452 | 0.6099 |
| HbA1c (%) > 7 (+/-) | 13/52 | 12/141 | 2.9375 | 1.2598 to 6.8495 | 0.0126 |
| HbA1c (%) > 7.5 (+/-) | 11/54 | 11/142 | 2.6296 | 1.0771 to 6.4199 | 0.0337 |
| HbA1c (%) > 8 (+/-) | 6/59 | 9/144 | 1.6271 | 0.5545 to 4.7747 | 0.3754 |
| Liver cirrhosis (+/-) | 40/25 | 96/57 | 0.9500 | 0.5226 to 1.7269 | 0.8664 |
| Liver cirrhosis Child-Pugh class B (+/-) | 6/59 | 12/141 | 1.1949 | 0.4283 to 3.3338 | 0.7337 |
| Serum albumin < 3 g/dL (+/-) | 3/62 | 3/150 | 2.4194 | 0.4752 to 12.3168 | 0.2873 |
| Hemoglobin < 10 g/dL (+/-) | 7/58 | 22/131 | 0.7187 | 0.2907 to 1.7764 | 0.4743 |
| CG-GFR (mL/min/1.73 m^2^) < 60 (+/-) | 13/52 | 60/93 | 0.3875 | 0.1946 to 0.7718 | 0.0070 |
| CG-GFR (mL/min/1.73 m^2^) < 45 (+/-) | 1/64 | 29/124 | 0.0668 | 0.0089 to 0.5017 | 0.0085 |
| AJCC tumor staging > III‡ (+/-) | 31/34 | 63/90 | 1.3025 | 0.7267 to 2.3347 | 0.3747 |
| (B) Factors within 3 days after TACE |  |  |  |  |  |
| Embolized tumor size > 5 cm (+/-) | 37/28 | 72/81 | 1.4866 | 0.8285 to 2.6675 | 0.1838 |
| TACE using DEMs (+/-) | 16/49 | 38/115 | 0.9882 | 0.5041 to 1.9371 | 0.9724 |
| Total IV fluid within 3 days after TACE < 1000 mL (+/-) | 3/62 | 7/146 | 1.0092 | 0.2527 to 4.0307 | 0.9896 |
| CG-GFR (mL/min/1.73 m^2^) < 60 patients treated with NAC§ (n = 73) (+/-) | 2/11 | 19/41 | 0.3923 | 0.0791 to 1.9469 | 0.2523 |

†: Four patients did not have serum creatinine data at the 3rd day after TACE. ‡: The American Joint Committee on Cancer (AJCC) TNM staging system 8th edition was applied for tumor staging. §: The patients took effervescent NAC 600 mg twice per day from day of admission till discharge. CI: confidence interval. CG-GFR: Cockcroft-Gault glomerular filtration rate, cTACE: conventional TACE, DEMs: drug-eluting microspheres, HbA1c: glycated hemoglobin, IV: intravenous, NAC: N-acetylcysteine, TACE: transarterial chemoembolization.
