# Supplementary material for: Safety of transarterial chemoembolization on renal function in combined hepatocellular carcinoma and chronic kidney disease patients
Source: Kaohsiung J Med Sci. 2024 Dec 30;41(2):e12925. doi: 10.1002/kjm2.12925 (PMC11827540; doi:10.1002/kjm2.12925)

Safety of transarterial chemoembolization on renal function in combined hepatocellular carcinoma and chronic kidney disease patients

Zu-Yau Lin*, Ming-Lun Yeh, Po-Cheng Liang, Chung-Feng Huang, Jee-Fu Huang, Chia-Yen Dai, Ming-Lung Yu, Wan-Long Chuang

Transarterial chemoembolization exerted same influence on renal function between patients with and without chronic kidney disease. Most patients showed improved renal function at discharge. Low serum albumin level, proteinuria and poor diabetes mellitus control were factors to exacerbate renal function after transarterial chemoembolization.

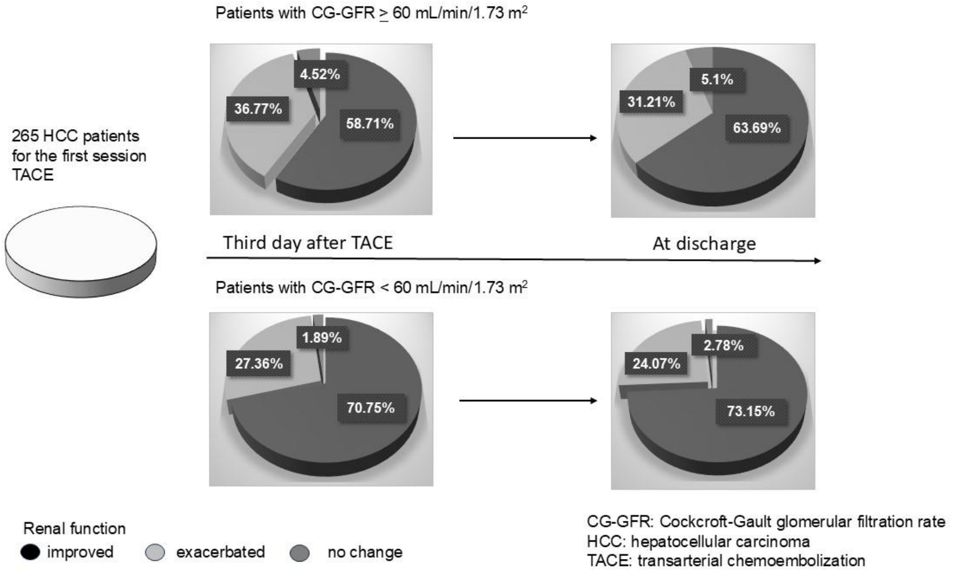

Supplement: Supplementary file 2 — Appendix S1 [file KJM2-41-e12925-s001.pdf]
